# Imaging-Based Pulmonary Hypertension Phenotypes in Connective Tissue Disease-Related Interstitial Lung Disease

**DOI:** 10.1016/j.chest.2026.02.045

**Published:** 2026-04-08

**Authors:** Sarah L. Khan, Kevin J. Psoter, Cheng Ting Lin, Aparna Balasubramanian, Catherine E. Simpson, Matthew R. Lammi, Tijana Tuhy, Rachel L. Damico, Todd M. Kolb, Paul M. Hassoun, Sonye K. Danoff, Stephen C. Mathai

**Affiliations:** a Division of Pulmonary and Critical Care Medicine, Johns Hopkins University School of Medicine, Baltimore, MD; b Division of General Pediatrics, Johns Hopkins University School of Medicine, Baltimore, MD; c Department of Radiology and Radiological Science, Johns Hopkins University School of Medicine, Baltimore, MD; d Department of Pulmonary and Critical Care Medicine, The Oregon Clinic, Portland, OR; e Department of Pulmonary, Critical Care, and Sleep Medicine, University of Miami Miller School of Medicine, Miami, FL

**Keywords:** clinical outcomes, CT, interstitial lung disease, phenotyping, pulmonary hypertension

## Abstract

**Background:**

Patients with connective tissue disease-related interstitial lung disease (CTD-ILD) may demonstrate pulmonary hypertension (PH) through pulmonary parenchymal destruction and chronic hypoxia or via endovascular changes driven by the underlying autoimmunity. Distinguishing which process—parenchymal or vascular—is the primary cause has important therapeutic and prognostic implications.

**Research Question:**

Can chest imaging be used to classify patients with CTD-ILD and PH systematically as having a parenchymal or vascular PH phenotype?

**Study Design and Methods:**

Chest CT imaging was used to classify 275 adult patients with CTD-ILD and PH in the Johns Hopkins ILD and PH registries as having a parenchymal or vascular PH phenotype. CT imaging-based PH phenotypes were determined using a validated ILD severity staging tool that involves visual inspection of the percentage of lung affected by CTD-ILD. The primary outcome was time to clinical worsening (defined as a composite of physiologic worsening, hospital admission for a pulmonary or cardiac diagnosis, lung transplantation, or death), which was compared between the 2 phenotypes. Secondary outcomes included comparisons of baseline cardiopulmonary testing results, time to physiologic worsening, time to hospital admission, and time to lung transplantation or death. Clinical outcomes also were compared between the CT imaging-based PH phenotypes and the conventional World Symposium on Pulmonary Hypertension (WSPH) group classifications.

**Results:**

Patients with CTD-ILD and the parenchymal PH phenotype were 1.76 times more likely to experience clinical worsening than those with the vascular phenotype. They were also more likely to experience physiologic worsening, hospitalization, and lung transplantation or death. CT imaging-based PH phenotypes were associated more closely with clinical outcomes than the WSPH group classifications.

**Interpretation:**

Our research shows that patients with CTD-ILD and the parenchymal PH phenotype have worse clinical outcomes than those with the vascular PH phenotype. CT imaging-based PH phenotypes may characterize patients’ risk of clinical worsening more reliably than the WSPH groups.

## Graphical Abstract

**Figure:**
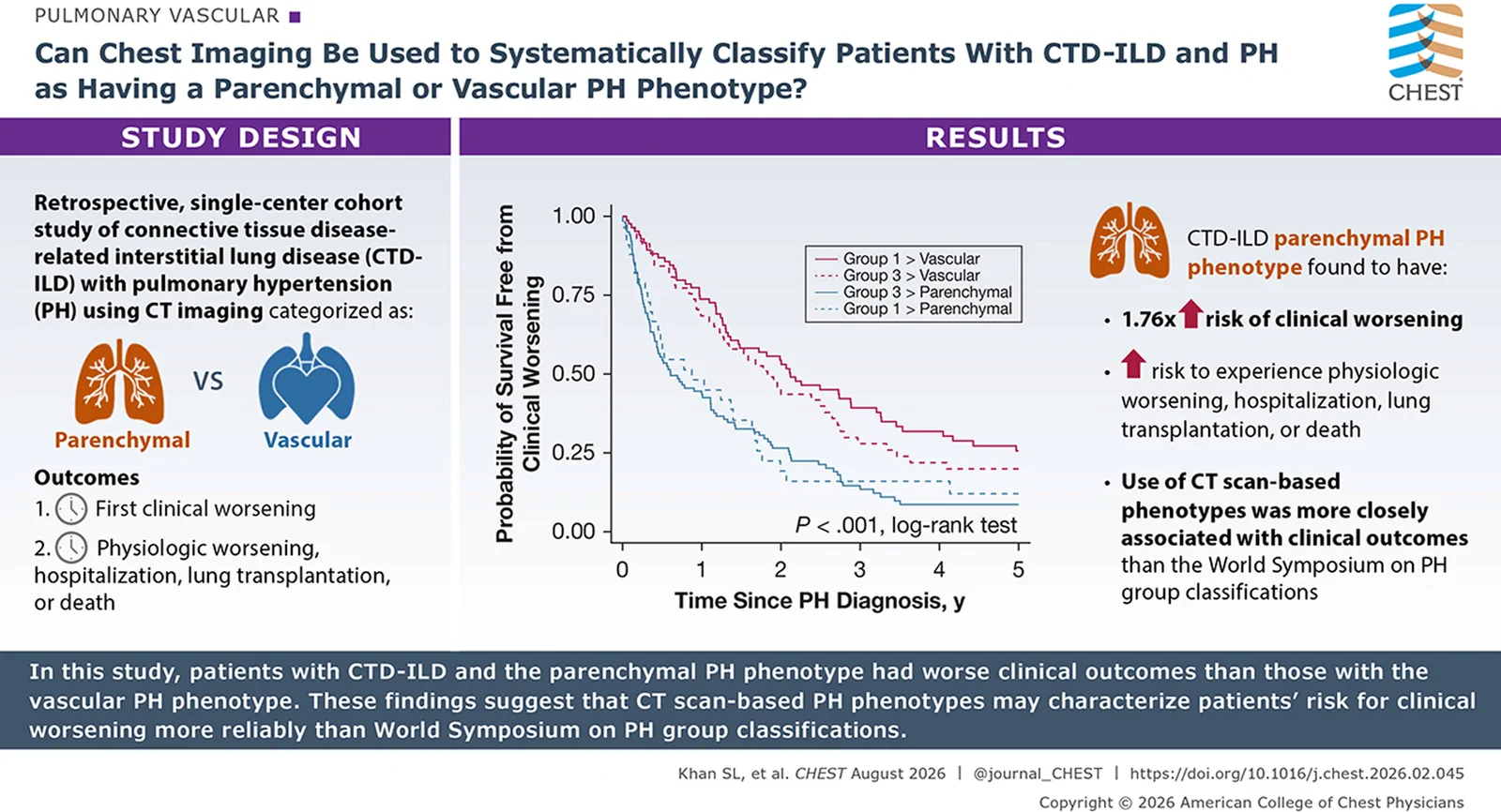

FOR EDITORIAL COMMENT, SEE PAGE 299

**Take-Home Points**

**Research Question:** Can chest imaging be used to classify patients with connective tissue disease-related interstitial lung disease (CTD-ILD) and pulmonary hypertension (PH) systematically as having a parenchymal or vascular PH phenotype?

**Results:** After systematic review of chest imaging, more than one-quarter of patients with CTD-ILD and PH in this cohort were reclassified from the clinically assigned WSPH group to the nonconcordant PH phenotype. These CT imaging-based PH phenotypes were associated more closely with clinical outcomes than the clinically assigned WSPH group diagnoses.

**Interpretation:** Our research shows that patients with CTD-ILD and PH can be classified using chest imaging as having a parenchymal or a vascular PH phenotype. These phenotypes seem to be associated with different clinical outcomes and may have clinical usefulness in guiding prognostication and therapeutic decisions for patients with CTD-ILD.

Pulmonary hypertension (PH) is a highly morbid and often fatal condition that affects approximately 25% of patients with connective tissue disease-related interstitial lung disease (CTD-ILD).^1^, ^2^, ^3^, ^4^, ^5^, ^6^, ^7^, ^8^ PH in CTD-ILD may arise principally from parenchymal injury with fibrotic ablation of pulmonary blood vessels and associated hypoxemia or from autoimmunity-induced pulmonary vascular endothelial dysfunction.^6^^,^^9^^,^^10^ Identifying the primary driver of the PH phenotype is a crucial step in the diagnostic process because their predicted clinical outcomes and therapeutic options can be quite different.^11^, ^12^, ^13^, ^14^, ^15^, ^16^, ^17^

The World Symposium on Pulmonary Hypertension (WSPH) defines 5 PH groups based on the underlying pathophysiologic and associated conditions.^17^, ^18^, ^19^ Group 3 PH is associated with chronic lung diseases such as CTD-ILD and is characterized by parenchymal destruction and aberrant remodeling of capillaries and venules. Group 1 PH, however, is characterized by endothelial dysfunction and smooth muscle cell proliferation in the pulmonary vasculature. Currently, to determine whether PH in individual patients with CTD-ILD is more consistent with group 1 or 3 disease, clinicians use pulmonary function testing (PFT) and chest imaging (ie, CT imaging) to compare subjectively the extent of pulmonary fibrosis with PH severity as assessed by right heart catheterization (RHC).^7^^,^^17^^,^^18^ Although current guidelines acknowledge these diagnostic dilemmas, no specific recommendations exist for distinguishing group 1 and 3 PH in patients with CTD-ILD.^17^^,^^20^ This leaves the critical determination of clinical classification to physician discretion. Given the WSPH groups’ significant differences in prognosis and treatment options, a pressing need exists for a standardized, reproducible, and readily accessible method for distinguishing PH phenotypes in patients with CTD-ILD.

To address this knowledge gap, we adapted a validated severity staging system based on chest CT scans and PFT results that was used previously to differentiate limited and extensive interstitial lung disease (ILD) phenotypes in scleroderma.^21^ In the current study, we applied this CT imaging-based system to patients with CTD-ILD and RHC-proven precapillary PH to classify the disease as having either a parenchymal phenotype analogous to group 3 PH or a vascular phenotype analogous to group 1 PH, and then compared the clinical features and outcomes.

## Study Design and Methods

### Study Population

We conducted a retrospective, single-center cohort study of adult patients with CTD-ILD who underwent RHC between January 1, 2003, and December 31, 2023, that fulfilled the 7th WSPH definition of precapillary PH.^17^^,^^22^ CTD-ILD diagnoses were confirmed by review of chest imaging, PFT results, clinical history documented by pulmonary and rheumatology specialists, and serologic testing. Patients with evidence of left-sided heart disease based on RHC hemodynamics, chronic thromboembolic disease (assessed via nuclear ventilation-perfusion scan or pulmonary angiography), or other systemic conditions associated with PH (eg, sarcoidosis, sickle cell disease and other hematologic conditions, end-stage renal disease, inborn errors of metabolism, etc.) were excluded from the study, as were patients who did not undergo chest CT imaging within 6 months before PH diagnosis.^17^ All patients provided informed consent to participate in either the Johns Hopkins Pulmonary Hypertension Center Registry (Identifier: IRB NA_00027124) or the Johns Hopkins Interstitial Lung Disease Registry (Identifier: IRB NA_00023365).

### Data Collection

Individual-level data was abstracted manually from patients’ electronic medical records. Demographic information included age at the time of PH diagnosis, sex, patient-reported race and ethnicity, smoking status, and connective tissue disease (CTD) diagnosis. RHC hemodynamics and baseline (obtained within 6 months of PH diagnosis) cardiopulmonary test results including PFT, 6-minute walk test, transthoracic echocardiography, and N-terminal pro-brain natriuretic peptide levels were recorded. The World Health Organization functional class at the time of PH diagnosis and physician-assigned WSPH group were documented along with any exposure to PH-specific or immunosuppressive medications after PH diagnosis.

For each patient, noncontrast chest CT imaging performed up to 6 months before PH diagnosis or the first available after diagnosis was selected for review. Chest CT imaging was performed during the inspiratory phase of respiration and in the supine position. Slice thickness was between 3 and 5 mm. The method for chest CT imaging review, which was adapted from the severity staging system for scleroderma-ILD developed by Goh et al,^21^ consisted of visual estimates of the percentage of lung involved by ground glass, reticulations, or honeycombing. Individuals with > 20% lung involvement on chest CT imaging were classified as having a parenchymal phenotype, whereas those with < 20% lung involvement were classified as having a vascular phenotype. No patients showed exactly 20% lung involvement, but if any had, FVC would have been used to distinguish the phenotypes. Additional details about these methods for chest CT imaging review are provided in e-Appendix 1 and e-Figure 1. Initially, and to minimize the introduction of bias resulting from reader variability, 100 chest CT scans were selected randomly and were reviewed by 2 independent readers (S. L. K. and C. T. L.) who were masked to the WSPH group classification. A preplanned threshold of 90% agreement was attained, and as a result, all remaining CT scans then were reviewed by a single reader (S. L. K.) who remained masked to patient WSPH group.

The primary outcome was the time to the first clinical worsening event, which was defined as a composite of physiologic worsening with a decline in FVC of ≥ 10%, 6-minute walk distance of ≥ 15%, hospital admission for a pulmonary or cardiac diagnosis, lung transplantation, or death resulting from any cause.^14^ Decline in FVC and 6-minute walk distance were included to account for competing risks of disease progression resulting from ILD or PH.^23^ Secondary outcomes included the times to physiologic worsening, hospital admission, and lung transplantation or death.

### Statistical Analysis

Demographic characteristics and baseline cardiopulmonary testing were summarized for patients with the parenchymal and vascular PH phenotypes and were compared using Fisher exact tests and Mann-Whitney *U* tests. Kaplan-Meier plots were produced for the primary and secondary outcomes and were compared using a log-rank test. Cohen’s κ statistics were used to compare patients’ classifications between CT imaging-based PH phenotypes and WSPH groups.

The association of selected individual-level characteristics with each outcome was evaluated using Cox proportional hazards regression. After confirming that the proportional hazards assumption was met, comparisons of the primary and secondary outcomes between the CT imaging-based parenchymal and vascular PH phenotypes were evaluated using unadjusted and multivariable Cox proportional hazards regression models. All adjusted models included age at the time of PH diagnosis, sex, patient-reported race, pulmonary vascular resistance (PVR), and FVC to diffusing capacity of the lungs for carbon monoxide (Dlco) ratio. For these analyses, patients entered risk sets at the time of PH diagnosis and were followed up until the time of the event, administrative censoring after 5 years, or December 31, 2024. The results of these models are presented as hazard ratios (HRs) with corresponding 95% CIs. A priori, comparisons of model fit and outcome discrimination were performed using Akaike’s information criteria and C statistics. A 2-sided *P* value of < .05 was considered statistically significant. All analyses used complete cases without imputation and were conducted using STATA version 18.0 software (StataCorp).

## Results

### PH Phenotypes and Baseline Characteristics

Two hundred seventy-five adult patients with CTD-ILD and precapillary PH were in the Johns Hopkins PH and ILD registries. Most were White female patients with scleroderma with a median age at the time at the time of PH diagnosis of 61 years (interquartile range [IQR], 49-68 years) (Table 1). PFT results showed moderate restriction (median FVC, 61% predicted [IQR, 47%-75% predicted]) with a severe reduction in Dlco (median, 36% predicted [IQR, 25%-47% predicted]). Functional capacity was impaired significantly with a median 6-minute walk distance of 284 m (IQR, 195-387 m). Hemodynamics showed moderate severity PH with a median PVR of 4.8 Wood units (IQR, 3.4-7.7 Wood units).

**Table 1:** Baseline Characteristics of the Cohort of Patients With CTD-ILD and PH Overall and by PH Phenotype

Characteristic	All (N = 275)	Parenchymal Phenotype (n = 134)	Vascular Phenotype (n = 141)
Age at PH diagnosis, y	60.8 (49.5-68.2)	60.4 (48.8-67.7)	61.4 (51.6-68.9)
Female sex	205 (74.6)	94 (70.2)	111 (78.7)
Patient-reported race			
White	144 (52.4)	65 (48.5)	79 (56.05)
Black	106 (38.6)	57 (42.5)	49 (34.8)
Asian	9 (3.3)	5 (3.7)	4 (2.8)
Other^a^	16 (5.8)	7 (5.2)	9 (6.4)
Ever smoking	129 (46.9)	59 (44.0)	70 (49.7)
CTD diagnosis			
Mixed connective tissue disease	21 (7.6)	11 (8.2)	10 (7.1)
Myositis	26 (9.5)	16 (11.9)	10 (7.1)
Rheumatoid arthritis	8 (2.9)	4 (3.0)	4 (2.8)
Scleroderma			
Limited	123 (45.2)	56 (42.1)	67 (48.2)
Diffuse	69 (25.4)	32 (24.1)	37 (26.6)
Systemic lupus erythematosus	5 (1.8)	3 (2.2)	2 (1.4)
Undifferentiated CTD	12 (4.4)	7 (5.2)	5 (3.6)
Overlap syndrome	20 (7.3)	9 (6.7)	11 (7.8)
Other CTD	2 (0.7)	2 (1.5)	0 (0.0)
PH diagnosis before 2013	99 (36.0)	48 (35.8)	51 (36.2)
PH medications			
Any PH medication	220 (80.0)	103 (76.9)	117 (83.0)
Phosphodiesterase type 5 inhibitor	199 (72.4)	92 (68.7)	107 (75.9)
Endothelin receptor antagonist	94 (34.2)	29 (21.6)	65 (46.1)
Prostacyclin			
Parenteral	8 (2.9)	1 (0.8)	7 (5.0)
Inhaled	46 (16.7)	21 (15.7)	25 (17.7)
Riociguat	1 (0.4)	1 (0.8)	0 (0.0)
Selexipag	3 (1.1)	1 (0.8)	2 (1.4)
Immunosuppression			
Any immunosuppression	216 (78.6)	112 (83.6)	104 (73.8)
Steroids	172 (62.6)	89 (66.4)	83 (58.9)
Antimetabolite	138 (50.2)	78 (58.2)	60 (42.6)
Calcineurin inhibitor	27 (9.8)	17 (12.7)	10 (7.1)
Hydroxychloroquine	11 (4.0)	7 (5.2)	4 (2.8)
Biologic	10 (3.6)	5 (3.7)	5 (3.6)
Other	26 (9.5)	15 (11.2)	11 (7.8)

Data are presented as No. (%) or median (interquartile range). CTD-ILD = connective tissue disease-related interstitial lung disease; PH = pulmonary hypertension.

a Multiracial or race not otherwise specified.

**Figure 1:**
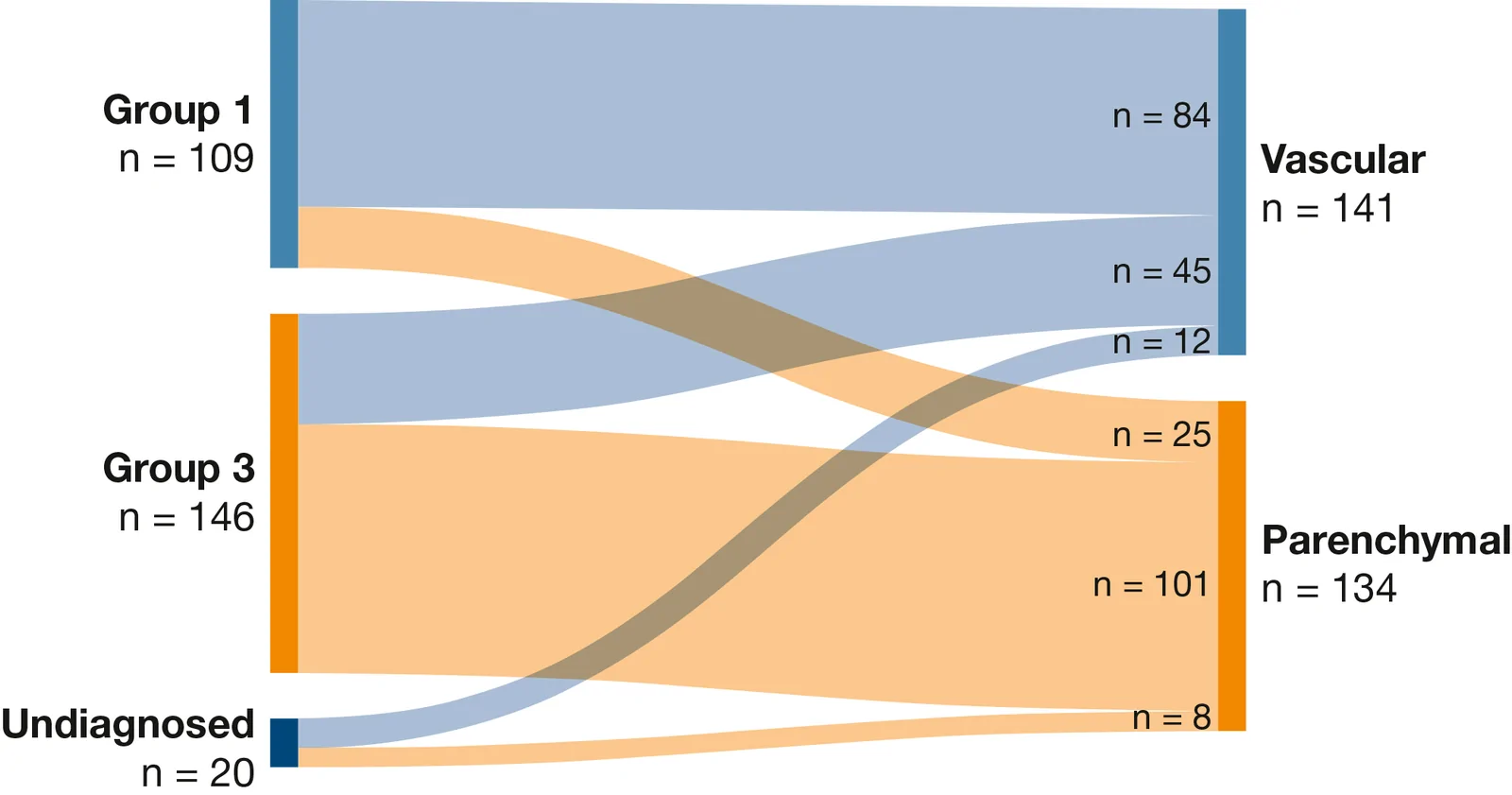
Sankey plot illustrating the reclassification of patients with connective tissue disease-related interstitial lung disease and pulmonary hypertension based on chest imaging findings.

One hundred nine patients (39.6%) received a diagnosis of WSPH group 1 PH and 146 patients (53.1%) received a diagnosis of WSPH group 3 PH from treating clinicians, and 20 patients (7.3%) did not receive a diagnosis of PH at the time of the RHC, but the hemodynamic findings met the current definition of precapillary PH. After reviewing the chest CT scans and PFT results of all patients with CTD-ILD and PH, 134 patients (48.7%) were classified as having a parenchymal PH phenotype and 141 patients (51.3%) were classified as having a vascular phenotype. Overall, 70 patients, 27.5% of those with a diagnosis of PH, were reclassified as having a CT imaging-based phenotype not concordant with the WSPH diagnosis (Cohen’s κ, 0.45; *P* < .01) (Fig 1). Patient characteristics by WSPH diagnosis and phenotype concordance are shown in e-Table 1.

### Baseline Cardiopulmonary Testing

At the time of PH diagnosis, patients with the parenchymal and vascular phenotypes showed similar hemodynamic features on diagnostic RHC (Table 2). Other measures of cardiac function, including transthoracic echocardiography parameters and N-terminal pro-brain natriuretic peptide levels, also were similar between the 2 phenotypes. Regarding baseline pulmonary function, patients with CTD-ILD and the parenchymal PH phenotype showed more severe restrictive ventilatory defects (FVC, 55% predicted vs 68% predicted; *P* < .01), more diffusion limitation (Dlco, 30% predicted vs 40% predicted; *P* < .01), lower carbon monoxide transfer coefficients (carbon monoxide transfer coefficient, 50% predicted vs 58% predicted; *P* < .01), and higher FVC to Dlco ratios (2.03 vs 1.85; *P* = .01) compared with patients with the vascular phenotype. During 6-minute walk testing, a greater proportion of patients with the parenchymal phenotype used supplemental oxygen (60% vs 36%; *P* < .01). Despite this, patients with the parenchymal phenotype showed lower nadir oxygen saturation (85% vs 88%; *P* < .01) and higher Borg dyspnea score (4 vs 3; *P* = .04). The actual distance walked was similar between the 2 phenotypes.

**Table 2:** Baseline Cardiac and Pulmonary Function Testing Results of Patients With CTD-ILD With the Parenchymal and Vascular PH Phenotypes

Variable	Parenchymal Phenotype (n = 134)	Vascular Phenotype (n = 141)	*P* Value
Right heart catheterization			
RAP, mm Hg	4 (2-7)	5 (3-8)	.16
mPAP, mm Hg	30 (25-39)	32 (25-43)	.15
mPAP 21-24 mm Hg	27 (20.1)	25 (17.7)	.65
PCWP, mm Hg	9 (6-11)	9 (6-11)	.89
CO, L/min	4.5 (3.6-5.4)	4.5 (3.5-5.5)	.89
PVR, WU	4.7 (3.5-7.3)	4.9 (3.4-8.7)	.52
TTE			
LVEF, %	60 (55-60)	60 (55-65)	< .01
Estimated RVSP, mm Hg	58 (49-74)	59 (41-73)	.26
RV dysfunction	60 (46.9)	54 (39.7)	.26
TAPSE, cm	1.78 (1.60-2.30)	2.00 (1.70-2.40)	.17
NT-pro-BNP, pg/mL	333 (97-2,268)	456 (170-1,471)	.43
Pulmonary function testing results			
FVC, % predicted	55 (43-68)	68 (53-79)	< .01
FEV_1_, % predicted	60 (45-71)	65 (53-80)	< .01
FEV_1_ to FVC ratio	0.83 (0.78-0.88)	0.80 (0.73-0.85)	< .01
Dlco, % predicted	30 (21-39)	40 (32-55)	< .01
KCO, % predicted	50 (39-64)	58 (49-76)	< .01
FVC to Dlco ratio	2.03 (1.67-2.52)	1.85 (1.25-2.27)	.01
6-minute walk testing results			
6MWD			
Meters	275 (183-373)	317 (233-396)	.08
% Predicted	54 (33-71)	59 (42-76)	.06
Supplemental oxygen use	70 (60.3)	44 (35.5)	< .01
Peripheral oxygen saturation at end of study	85 (79-89)	88 (83-93)	< .01
Borg dyspnea score at end of study	4 (3-5)	3 (2-4)	.03
WHO functional class 3 or 4	85 (69.7)	77 (55.8)	.03

Data are presented as No. (%) or median (interquartile range) unless otherwise indicated. 6MWD = 6-minute walk distance; CO = cardiac output; CTD-ILD = connective tissue disease-related interstitial lung disease; Dlco = diffusing capacity of the lungs for carbon monoxide; KCO = carbon monoxide transfer coefficient; LVEF = left ventricular ejection fraction; mPAP = mean pulmonary artery pressure; NT-pro-BNP = N-terminal pro-brain natriuretic peptide; PCWP = pulmonary capillary wedge pressure; PH = pulmonary hypertension; PVR = pulmonary vascular resistance; RAP = right atrial pressure; RVSP = right ventricle; RVSP = right ventricular systolic pressure; TAPSE = tricuspid annular plane systolic excursion; TTE = transthoracic echocardiography; WHO = World Health Organization; WU = Wood unit.

### Time to Clinical Worsening

**Figure 2:**
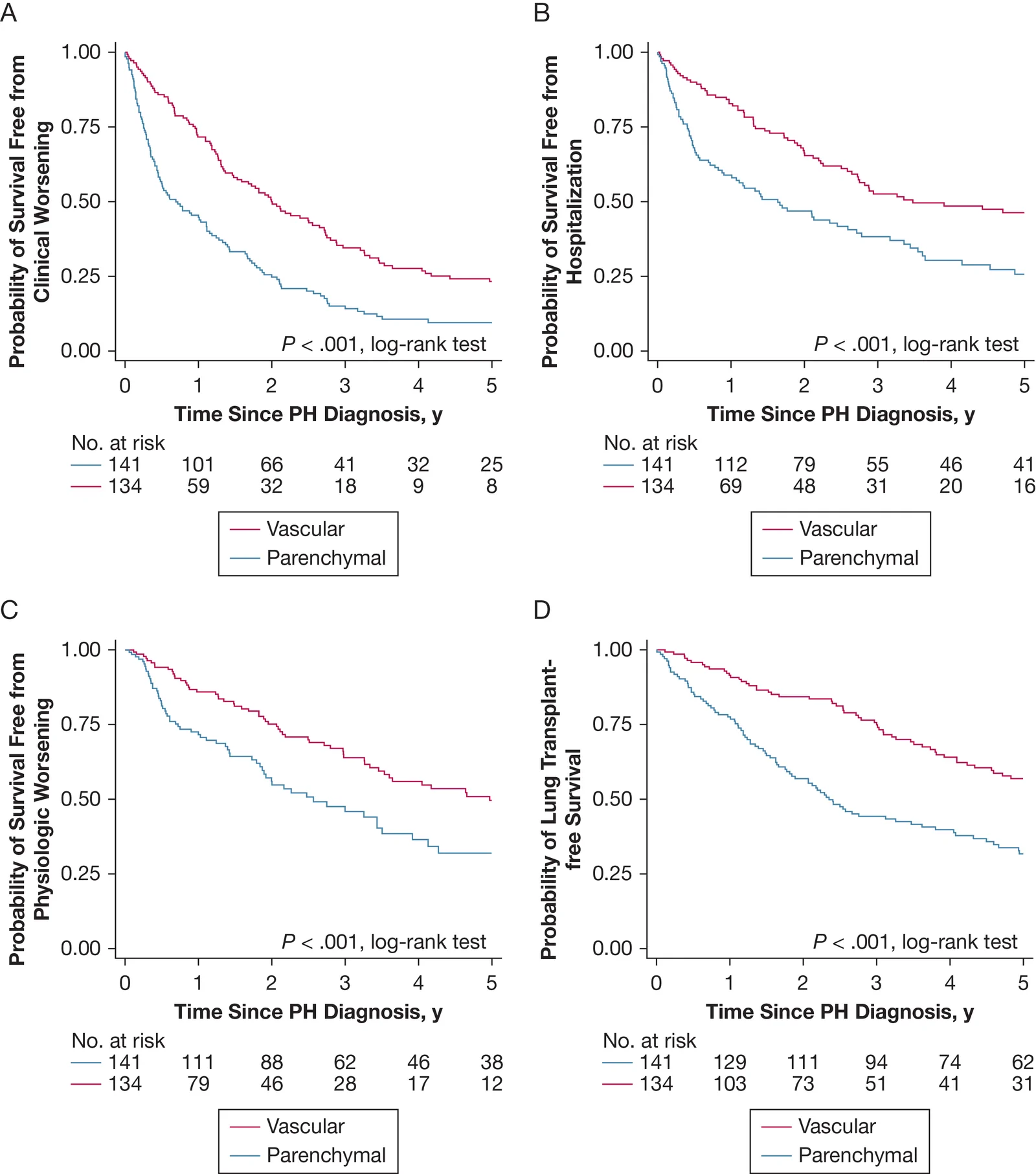
A-D, Kaplan-Meier curves for the primary outcome of clinical worsening (A) and secondary outcomes of hospitalization for a cardiac or pulmonary diagnosis (B), physiologic worsening (C), and transplant-free survival (D) by phenotype. PH = pulmonary hypertension.

One hundred eighteen patients with CTD-ILD and the parenchymal PH phenotype (88.1%) and 102 patients (72.3%) with CTD-ILD and the vascular PH phenotype experienced a clinical worsening event in the 5 years after the PH diagnosis (Table 3). The median time to clinical worsening was 0.69 years (95% CI, 0.45-1.11 years) for patients with the parenchymal phenotype and 2.00 years (95% CI, 1.48-2.65 years) for those with the vascular phenotype (*P* < .01). Kaplan-Meier curves for the primary and secondary outcomes are shown in Figure 2. Patient characteristics and baseline physiologic measures associated with clinical worsening are shown in e-Table 2.

**Table 3:** Five-Year Incidence Rates and Cox Proportional Hazards Ratios for the Primary and Secondary Outcomes in Patients With CTD-ILD Comparing the Parenchymal and Vascular PH Phenotypes

Variable	Parenchymal (n = 134)	Vascular (n = 141)	HR (95% CI)	
			Unadjusted	Adjusted
Clinical worsening	118 (88.1)	102 (72.3)	1.98 (1.51-2.58)	1.76 (1.27-2.44)
Hospitalization	82 (61.2)	66 (46.8)	1.90 (1.37-2.64)	1.71 (1.15-2.54)
Physiologic worsening	62 (46.3)	56 (39.7)	1.84 (1.28-2.65)	1.80 (1.18-2.74)
Death or lung transplantation	86 (64.2)	55 (39.0)	2.28 (1.63-3.21)	2.32 (1.51-3.57)

Data are presented as No. (%) unless otherwise indicated. Models adjusted for age at the time of PH diagnosis, sex, race, pulmonary vascular resistance, and baseline FVC to Dlco ratio. CTD-ILD = connective tissue disease-related interstitial lung disease; Dlco = diffusing capacity of the lungs for carbon monoxide; HR = hazard ratio; PH = pulmonary hypertension.

After adjusting for age at the time of PH diagnosis, sex, patient-reported race, PVR, and baseline FVC to Dlco ratio, patients with CTD-ILD and the parenchymal PH phenotype showed a higher risk for clinical worsening (HR, 1.76; 95% CI, 1.27-2.44) compared with those with the vascular phenotype (Table 3). Patients with the parenchymal phenotype showed similarly increased risks for hospitalization (HR, 1.71; 95% CI, 1.15-2.54), physiologic worsening (HR, 1.80; 95% CI, 1.18-2.74), and lung transplantation or death (HR, 2.32; 95% CI, 1.51-3.57). Because of the predominance of scleroderma in the cohort, we conducted a sensitivity analysis that showed similar risks for clinical worsening and its components based on CT imaging-based PH phenotype for patients with scleroderma-associated CTD and non-scleroderma-associated CTD, although these results were not statistically significant in the smaller cohort of patients with non-scleroderma-associated CTD (e-Table 3).

### PH Medication and Immunosuppression Exposure

Most patients with CTD-ILD and PH—103 patients (76.9%) with the parenchymal phenotype and 117 patients (83.0%) with the vascular phenotype—were exposed to PH medications. The most frequently prescribed medications were phosphodiesterase type 5 inhibitors followed by endothelin receptor antagonists (Table 1). No statistically significant associations were found between PH medications and the risk of clinical worsening, hospitalization, physiologic worsening, lung transplantation, or death for patients with either phenotype (e-Table 4). The risk for each of these clinical outcomes remained higher for patients with the parenchymal phenotype who received PH medications compared with those with the vascular phenotype (e-Table 5).

One hundred twelve patients (83.6%) with CTD-ILD and the parenchymal PH phenotype and 104 patients (73.8%) with CTD-ILD and the vascular phenotype received an immunosuppressive medication. Patients with CTD-ILD and the parenchymal PH phenotype who received immunosuppressants showed an increased risk for hospitalization, but not clinical worsening, physiologic worsening, lung transplantation, or death. No differences were found in the primary or secondary outcomes for patients with vascular phenotype who received immunosuppression compared with those who did not (e-Table 4). The risk for each outcome remained higher for patients with the parenchymal PH phenotype who were exposed to immunosuppressive medication compared with those with the vascular phenotype (e-Table 5).

### Comparing Phenotypes and WSPH Groups

**Figure 3:**
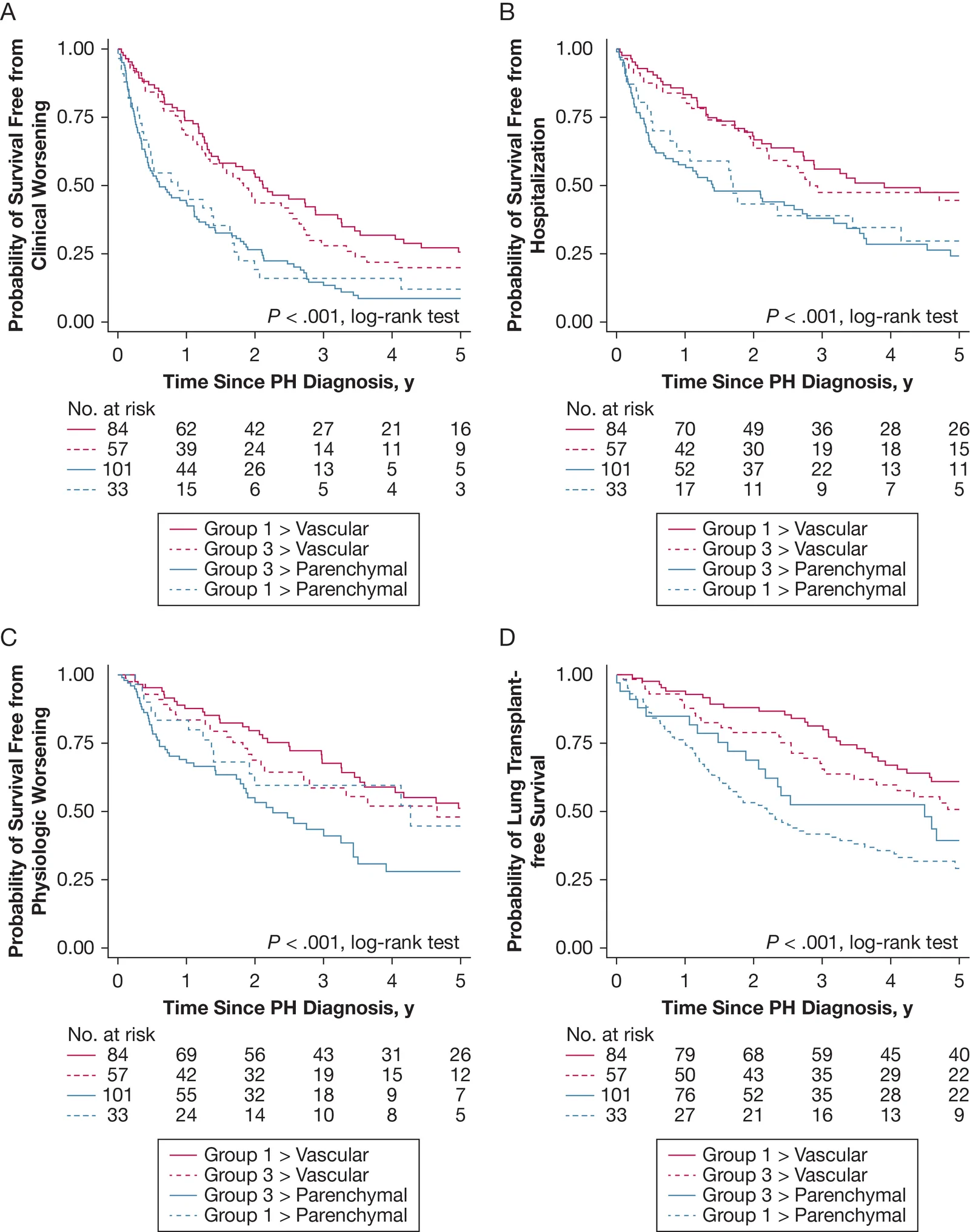
A-D, Kaplan-Meier curves comparing phenotypes by WSPH group concordance for the primary outcome of clinical worsening (A) and secondary outcomes of hospitalization for a cardiac or pulmonary diagnosis (B), physiologic worsening (C), and transplant-free survival (D). PH = pulmonary hypertension; WSPH = World Symposium on Pulmonary Hypertension.

Patients classified as having WSPH group 3 PH and a concordant parenchymal PH phenotype showed a greater probability of clinical worsening than patients with concordant WSPH group 1 PH diagnoses and vascular PH phenotypes (HR, 2.05; 95% CI, 1.32-3.16). Patients with discordant WSPH diagnoses and CT imaging-based PH phenotypes—that is, patients who had received a diagnosis of WSPH group 1 PH, but whose chest CT scans indicated a parenchymal PH phenotype, and vice versa—were compared with the patients with concordant WSPH group diagnoses and phenotypes. The Kaplan-Meier curves for the patients with discordant WSPH diagnoses and PH phenotypes closely followed the curves representing patients with the same CT imaging-based phenotype for clinical worsening and hospitalization and fell between the curves for physiologic worsening and lung transplant-free survival (Fig 3).

When comparing the model fit of the Cox proportional hazards models for the primary and secondary outcomes based on CT imaging-based PH phenotypes and WSPH group diagnoses, the phenotype-based models showed slightly better fit with lower Akaike’s information criteria. The CT imaging-based PH phenotypes also yielded models with minimally improved C statistics for each of the primary and secondary outcomes compared with the WSPH groups (e-Table 6).

## Discussion

In this study, we adapted a chest CT imaging-based ILD severity staging system to classify patients with CTD-ILD and PH systematically as having either a parenchymal or vascular phenotype to address a gap in the current approach to diagnosing this disease according to the canonical WSPH groups. Patients with CTD-ILD and the parenchymal PH phenotype compared with those with CTD-ILD and the vascular phenotype showed worse clinical outcomes in the 5 years after the PH diagnosis regarding clinical worsening, hospitalization for a pulmonary or cardiac diagnosis, physiologic worsening, and lung transplantation or death. Importantly, when we compared patient classifications based on WSPH group diagnoses and the CT imaging-based phenotypes, more than one-quarter were reclassified with the nonconcordant phenotype based on the extent of parenchymal lung disease seen on chest CT imaging. Patients with discordant WSPH group diagnoses and CT imaging-based phenotypes showed clinical worsening and hospitalization rates more similar to patients with the same CT imaging-based phenotype and whose WSPH groups and phenotypes were concordant, whereas their rates of physiologic worsening and lung transplantation were between those of the patients with concordant WSPH group diagnoses and CT imaging-based PH phenotypes.

The finding that patients with CTD-ILD and the parenchymal PH phenotype showed worse clinical outcomes than those with the vascular phenotype was expected based on previous studies using WSPH classification in various CTD and PH cohorts.^13^^,^^24^^,^^25^ Most recently, work from our group demonstrated significant survival differences for patients with scleroderma with pulmonary arterial hypertension compared with those with PH related to ILD (PH-ILD), with median survival times of 8.8 years and between 3 and 4 years, respectively.^13^ Similarly, recent studies in populations with idiopathic pulmonary arterial hypertension have found worse outcomes for patients with a so-called lung disease phenotype, defined by smoking history and low Dlco or by qualitative assessment of chest CT scans with mild, moderate, or severe parenchymal lung involvement.^26^^,^^27^ Together, these studies highlight the association of varying degrees of lung disease on outcomes across the spectrum of precapillary PH and provide motivation for the current study.

One intriguing aspect of our study is the higher FVC to Dlco ratio observed in patients with CTD-ILD with the parenchymal PH phenotype compared with those with the vascular phenotype. Previous studies, particularly in patients with scleroderma, have shown that a lower Dlco % predicted and thus higher FVC to Dlco ratio is associated strongly with the presence of PH in patients with and without ILD and may favor a diagnosis of pulmonary arterial hypertension over PH-ILD.^4^^,^^28^, ^29^, ^30^ Physiologically, we might expect that the vascular PH phenotype would have a reduced Dlco % predicted out of proportion to the degree of restriction measured by FVC % predicted, whereas these would be proportionally reduced in the parenchymal phenotype. The deviation of the present cohort from previous observations may be explained in part by the inclusion of non-scleroderma-associated CTDs that have not been well characterized in this context. Furthermore, evidence from patients with idiopathic pulmonary fibrosis on the lung transplant waiting list shows that the correlation between the rate of change in FVC and PVR is complex, changes over time, and depends on baseline pulmonary function.^31^ This may indicate that the complex molecular and physiologic influences of the pulmonary epithelium and vascular endothelium on each other vary depending on the baseline disease state and changes in lung function over time.^32^, ^33^, ^34^, ^35^, ^36^, ^37^ These findings warrant further investigation in a prospective, well-phenotyped cohort with serial RHC and PFT measurements.

The novel aspects of this study lie in the use of a systematic approach to classify PH in patients with CTD-ILD based on the extent of lung involvement. Our results show that application of this methodology distinguishes vascular and parenchymal phenotypes of PH in patients with CTD-ILD that seem to predict risk for clinical worsening and hospitalization better than the more subjective WSPH group diagnoses. Interestingly, patients with nonconcordant WSPH group diagnoses and CT imaging-based PH phenotypes seem to have intermediate risks for physiologic worsening and lung transplantation or death, which may be an indication of a third mixed phenotype, rather than the dichotomous diagnoses or phenotypes that have been described and investigated to date. This may have important implications for clinical care because the 2 phenotypes, which are analogous to WSPH group 1 and 3 PH, have different disease trajectories and treatment options. Therefore, by improving clinicians’ ability to classify PH objectively and accurately in patients with CTD-ILD, they may advise patients better regarding the expected clinical courses and appropriate therapies. Importantly, this methodology is simple; does not require radiologic expertise, additional software, or artificial intelligence applications; and has excellent agreement between readers. Regarding potential research applications, the availability of a more objective method for differentiating the parenchymal and vascular PH phenotypes could reduce heterogeneity among clinical trial participants and could improve the efficiency and quality of future trials of PH therapies.

To that end, we conducted an exploratory analysis comparing the clinical outcomes of patients with the parenchymal and vascular PH phenotypes who received medications for PH or ILD. This was motivated by the limited options for treating PH-ILD. Currently, only 1 medication, inhaled treprostinil, has demonstrated a clinical benefit for patients with PH-ILD.^14^ Interestingly, patients with CTD-ILD seem to have significant functional improvements when treated with inhaled treprostinil compared with other ILD diagnoses.^14^ Trials of other PH medications for patients with ILD with and without PH have either yielded no clinical benefit or have caused harm, but most have not been investigated specifically in the unique context of CTD-ILD and PH.^38^, ^39^, ^40^, ^41^, ^42^, ^43^, ^44^, ^45^ Although our study did not find any association between PH or ILD therapies and clinical outcomes for either phenotype, the analysis was conducted retrospectively with limited information about the duration and timing of PH or ILD medication exposures or adverse medication effects and was subject to confounding by indication. These limitations highlight the imperative need for a prospective study of phenotype-specific PH therapies for patients with CTD-ILD and the parenchymal and vascular PH phenotypes.

This study has several limitations. First, although the CT imaging-based severity staging system has been validated in CTD-ILD, this application to patients with CTD-ILD and PH is novel, occurred at a single center, and needs to be evaluated further in an external cohort. Second, the patients with CTD-ILD in this study may have been referred for PH evaluation by a primary pulmonologist, rheumatologist, or a provider outside of our hospital system, so no standardized approach to screening patients for PH was followed. As such, it is possible that ascertainment bias toward earlier PH screening for patients with one phenotype or another that might introduce lead-time bias into the reported time-to-event analyses may be present but unaccounted for. Third, the patients with CTD-ILD included in this study received a diagnosis of PH over a 20-year period. This time span was necessary to capture a large enough cohort to study the intersection of these rare diseases, but may have introduced confounding because of changes in the criteria used to diagnose PH and the standards of care for treating PH over that time. Fourth, we have reported that there seems to be no effect of exposure to a PH medication or immunosuppression on the primary and secondary outcomes, but the small number of patients who were not exposed to medication limited the power of these analyses. Finally, three-quarters of the patients in this study had scleroderma. As such, it is possible that our findings are most applicable to patients with scleroderma-related ILD and PH and less so to patients with other CTD diagnoses. The sensitivity analysis that we conducted was underpowered to address this question adequately, which will require further investigation, ideally in a multicenter prospective study.

Several avenues exist for future investigation. Of highest priority is prospective validation of these findings in an external cohort of patients with CTD-ILD and PH. Second, because significant differences exist in the approaches to treating WSPH group 1 and 3 disease, it will also be important to understand the effect of guideline-concordant PH therapy on clinical outcomes for patients with CTD-ILD and the parenchymal and vascular PH phenotypes. Finally, because currently no guidelines exist for screening patients with CTD-ILD for PH, investigations focused on changes in chest imaging as a predictor of PH developing may be informative.

## Interpretation

This study demonstrated that chest imaging can be used systematically to classify patients with CTD-ILD and PH as having a parenchymal or vascular PH phenotype. Using this system, more than one-quarter of patients in this cohort were reclassified from their clinically assigned WSPH group. Patients with CTD-ILD and the parenchymal PH phenotype showed worse clinical outcomes compared with those with the vascular phenotype. Application of this system to patients with CTD-ILD and PH offers a novel standardized approach to classification to inform treatment and prognosis in this vulnerable population. Patients with CTD-ILD and PH can be classified using chest imaging as having a parenchymal or a vascular PH phenotype. These phenotypes seem to be associated with different clinical outcomes and may have clinical usefulness in guiding prognostication and therapeutic decisions for patients with CTD-ILD.

## Funding/Support

This work was supported by the 10.13039/100000050National Heart, Lung, and Blood Institute [Grants T32HL007534 (S. L. K.) and F32HL170482 (S. L. K.)], the Pulmonary Fibrosis Foundation Scholars Program (S. L. K.), and the Dana Kusch Fund for PH-ILD Research at Johns Hopkins (S. L. K., S. K. D., and S. C. M.). A. B. is supported by the National Heart, Lung, and Blood Institute. C. E. S is supported by the National Heart, Lung, and Blood Institute and the Pulmonary Hypertension Association. R. L. D. is supported by the National Heart, Lung, and Blood Institute and the American Lung Association. P. M. H. is supported by the 10.13039/100000050National Heart, Lung, and Blood Institute.

## Financial/Nonfinancial Disclosures

The authors have reported to *CHEST* the following: S. L. K. reports honoraria from the American College of Chest Physicians. M. R. L. reports research support to institution from United Therapeutics and Avalyn Pharmaceuticals. R. L. B. reports honoraria from National Jewish Health. T. M. K. is the chief medical officer for Oxiwear, Inc. P. M. H. is a consultant for Merck & Co. and Aria CV. S. K. D. is the site primary investigator for studies sponsored by Bristol Myers Squibb, Boehringer-Ingelheim, and Genentech/Roche; receives royalties from UpToDate; is a consultant for Boehringer-Ingelheim, Lupin Pharma, and BMS; reports honoraria from the France Foundation; serves on the data safety and monitoring board of the Galecto Galactic Trial and Galapagos ISABELA Trial; is the senior medical advisor to the Pulmonary Fibrosis Foundation; and serves on the board of directors of the American Thoracic Society. S. C. M. reports research support from the Department of Defense and serves on the data safety and monitoring board of Janssen, United Therapeutics, Merck, and Acceleron. None declared (K. J. P., C. T. L., A. B., C. E. S., T. T.).

## Supplementary Data

e-Online Data
