# Supplementary material for: Imaging-Based Pulmonary Hypertension Phenotypes in Connective Tissue Disease-Related Interstitial Lung Disease
Source: Chest. 2026 Apr 8;170(2):499–511. doi: 10.1016/j.chest.2026.02.045 (PMC13494247; doi:10.1016/j.chest.2026.02.045)
Supplement: Supplementary file 1 [file mmc1.docx]

**Supplemental Materials**

*Method for Chest CT Review*

The method for chest CT review was adapted from the severity staging system for scleroderma-ILD developed by Goh, *et al*.^21^ Visual assessments of the percent of lung affected by interstitial thickening, ground glass, or honeycombing were made at five anatomic levels – (1) the aortic arch, (2) the main carina, (3) the inflow of the right pulmonary vein into the left atrium, (4) halfway between levels 3 and 5, and (5) the top of the right hemidiaphragm – and rounded to the nearest five percent. An average was then taken of the percent of lung involved at all five levels; if greater than 20%, the patient was classified as having extensive lung disease with a parenchymal PH phenotype, and if less than 20%, they were classified as having limited lung disease with a vascular phenotype. If the average lung involvement was exactly 20%, the FVC was used to make the determination with a reduced FVC less than 70% predicted indicating a parenchymal PH phenotype and a preserved FVC greater than or equal to 70% predicted indicating a vascular PH phenotype.

*Rationale for Cox Proportional Hazards Model Covariate Selection*

All of the multivariate Cox proportional hazards models were adjusted for age at the time of PH diagnosis, sex, patient-reported race, pulmonary vascular resistance (PVR), and the ratio of the percent predicted forced vital capacity to diffusing capacity of the lungs for carbon monoxide (FVC/DLCO). Age, sex, patient-reported race, and PVR were included based on clinical rationale and prior study data that indicates that PVR is strongly associated with clinical outcomes in patients with pulmonary arterial hypertension.^36^ Baseline FVC/DLCO was included based on its association with the primary and secondary outcomes for this study as shown e-Table 2 which shows the results of the univariate Cox proportional hazards ratios for demographic and clinical characteristics.

e-Figure 1. Chest CT-based phenotyping system for PH in patients with CTD-ILD. Adapted from the severity staging system for scleroderma-ILD developed by Goh, *et al.*^20^

e-Table 1. Baseline characteristics and cardiopulmonary testing of patients with CTD-ILD with the parenchymal and vascular PH phenotypes by WSPH group and phenotype concordance. All measured and calculated values are presented as median (IQR). Proportions of individuals are presented as N (%).

|  | **Parenchymal**  (N = 134) | | | **Vascular**  (N = 141) | | |
| --- | --- | --- | --- | --- | --- | --- |
|  | **Concordant**  (N = 101) | **Non-**  **concordant**  (N = 25) | **Undiagnosed**  (N = 8) | **Concordant**  (N = 84) | **Non-**  **concordant**  (N = 45) | **Undiagnosed**  (N = 12) |
| Age at PH diagnosis (years) | 58.2 (17.3) | 64.7 (17.9) | 68.3 (9.0) | 63.1 (19.6) | 58.6 (11.6) | 53.2 (14.9) |
| Female sex (N, %) | 69 (68.3%) | 25 (100%) | 7 (87.5%) | 67 (79.8%) | 34 (75.6%) | 10 (83.3%) |
| Patient-reported race (N, %) |  |  |  |  |  |  |
| White | 47 (46.5%) | 16 (64.0%) | 2 (25.0%) | 47 (56.0%) | 26 (57.8%) | 6 (50.0%) |
| Black | 45 (44.6%) | 7 (28.0%) | 5 (62.5%) | 28 (33.3%) | 16 (35.6%) | 5 (41.7%) |
| Asian | 5 (5.0%) | 0 (0.0%) | 0 (0.0%) | 3 (3.6%) | 1 (2.2%) | 0 (0.0%) |
| Other | 4 (4.0%) | 2 (8.0%) | 1 (12.5%) | 6 (7.1%) | 2 (4.4%) | 1 (8.3%) |
| Ever smoker (N, %) | 43 (42.6%) | 13 (52.0%) | 3 (37.5%) | 43 (51.2%) | 20 (44.4%) | 7 (58.3%) |
| CTD diagnosis (N, %) |  |  |  |  |  |  |
| Mixed connective tissue disease | 8 (7.9%) | 2 (8.0%) | 1 (12.5%) | 8 (9.5%) | 2 (4.4%) | 0 (0.0%) |
| Myositis | 13 (12.9%) | 2 (8.0%) | 1 (12.5%) | 2 (3.4%) | 5 (11.1%) | 3 (25.0%) |
| Rheumatoid arthritis | 4 (4.0%) | 0 (0.0%) | 0 (0.0%) | 2 (2.4%) | 2 (4.4%) | 0 (0.0%) |
| Sclerodema, limited | 36 (36.0%) | 15 (60.0%) | 5 (62.5%) | 46 (55.4%) | 15 (34.1%) | 6 (50.0%) |
| Scleroderma, diffuse | 28 (28.0%) | 4 (16.00%) | 0 (0.0%) | 18 (21.7%) | 16 (36.4%) | 3 (25.0%) |
| Systemic lupus erythematosus | 5 (5.0%) | 1 (4.0%) | 1 (12.5%) | 1 (1.2%) | 4 (8.9%) | 0 (0.0%) |
| Undifferentiated CTD | 7 (6.9%) | 2 (8.0%) | 0 (0.0%) | 9 (10.7%) | 1 (2.2%) | 1 (8.3%) |
| Overlap syndrome | 2 (2.0%) | 0 (0.0%) | 0 (0.0%) | 0 (0.0%) | 0 (0.0%) | 0 (0.0%) |
| Other CTD | 0 (0.0%) | 0 (0.0%) | 0 (0.0%) | 0 (0.0%) | 0 (0.0%) | 0 (0.0%) |
| PH diagnosis before 2013 (N, %) | 33 (32.7%) | 11 (44.0%) | 4 (50.0%) | 30 (35.7%) | 17 (37.8%) | 4 (33.3%) |
| PH medications |  |  |  |  |  |  |
| Any PH medication | 77 (76.2%) | 22 (88.0%) | 4 (50.0%) | 77 (91.7%) | 32 (71.1%) | 8 (66.7%) |
| PDE5-inhibitor | 68 (67.3%) | 20 (80.0%) | 4 (50.0%) | 72 (85.7%) | 28 (62.2%) | 7 (58.3%) |
| Endothelin receptor antagonist | 12 (11.9%) | 15 (60.0%) | 2 (25.0%) | 51 (60.7%) | 13 (28.9%) | 1 (8.3%) |
| Prostacyclin, parenteral | 0 (0.0%) | 1 (4.0%) | (0.0%) | 3 (3.6%) | 2 (4.4%) | 2 (16.7%) |
| Prostacyclin, inhaled | 17 (16.8%) | 3 (12.0%) | 1 (12.5%) | 16 (19.1%) | 8 (17.8%) | 1 (8.3%) |
| Riociguat | (0.0%) | 1 (4.0%) | (0.0%) | (0.0%) | (0.0%) | (0.0%) |
| Selexipag | 1 (1.0%) | (0.0%) | (0.0%) | 1 (1.2%) | 1 (2.2%) | (0.0%) |
| Immunosuppression |  |  |  |  |  |  |
| Any immunosuppression | 90 (89.1%) | 18 (72.0%) | 4 (50.0%) | 58 (69.1%) | 37 (82.2%) | 9 (75.0%) |
| Steroids | 72 (71.3%) | 13 (52.0%) | 4 (50.0%) | 43 (51.2%) | 32 (71.1%) | 8 (66.7%) |
| Antimetabolite | 64 (63.4%) | 12 (48.0%) | 2 (25.0%) | 35 (41.7%) | 18 (40.0%) | 7 (58.3%) |
| Calcineurin inhibitor | 15 (14.9%) | 2 (8.0%) | 0 (0.0%) | 5 (6.0%) | 4 (8.9%) | 1 (8.3%) |
| Hydroxychloroquine | 5 (5.0%) | 2 (8.0%) | 0 (0.0%) | 2 (2.4%) | 2 (4.4%) | 0 (0.0%) |
| Biologic | 3 (3.0%) | 2 (8.0%) | 0 (0.0%) | 0 (0.0%) | 3 (6.7%) | 2 (16.7%) |
| Other | 13 (12.9%) | 2 (8.0%) | 0 (0.0%) | 4 (4.8%) | 4 (8.9%) | 3 (25.0%) |
| Right heart catheterization |  |  |  |  |  |  |
| RAP (mmHg) | 4 (5) | 6 (4) | 4 (6) | 5 (5) | 5 (6) | 5 (4) |
| mPAP (mmHg) | 30 (12) | 32 (12) | 22 (2) | 36 (20) | 30 (16) | 24 (2) |
| mPAP 21-24 mmHg (N, %) | 15 (14.9%) | 4 (16.0%) | 8 (100%) | 4 (4.8%) | 9 (20.0%) | 12 (100%) |
| PCWP (mmHg) | 8 (5) | 10 (3) | 8 (4) | 9 (5) | 9 (5) | 7 (5) |
| CO (L/min) | 4.6 (1.8) | 4.4 (0.9) | 3.6 (1.4) | 4.5 (1.9) | 4.5 (2.0) | 4.7 (1.9) |
| PVR (WU) | 4.7 (3.5) | 5.2 (7.2) | 3.8 (2.8) | 6.5 (6.3) | 4.3 (3.9) | 3.4 (1.6) |
| TTE |  |  |  |  |  |  |
| LVEF (%) | 58 (5) | 60 (10) | 55 (5) | 60 (10) | 60 (10) | 60 (10) |
| Estimated RVSP (mmHg) | 57 (25) | 61 (29) | 60 (51) | 62 (32) | 59 (26) | 42 (16) |
| RV dysfunction (N, %) | 43 (45.3%) | 12 (48.0%) | 5 (62.5%) | 33 (40.2%) | 18 (41.9%) | 3 (27.3%) |
|  | **Parenchymal**  (N = 134) | | | **Vascular**  (N = 141) | | |
|  | **Concordant**  (N = 101) | **Non-**  **concordant**  (N = 25) | **Undiagnosed**  (N = 8) | **Concordant**  (N = 84) | **Non-**  **concordant**  (N = 45) | **Undiagnosed**  (N = 12) |
| TAPSE (cm) | 1.7 (0.6) | 2.6 (0.7) | 1.7 (1.0) | 2.1 (0.8) | 1.9 (0.4) | 2.5 (0.4) |
| Pro-BNP (pg/mL) | 271 (2179) | 484 (1052) | 2030 (5165) | 580 (2272) | 360 (655) | 205 (300) |
| Pulmonary function tests |  |  |  |  |  |  |
| FVC % predicted | 54 (22) | 65 (22) | 41 (29) | 75 (26) | 57 (29) | 68 (38) |
| FEV_1_ % predicted | 57 (23) | 68 (18) | 48 (35) | 73 (25) | 56 (27) | 56 (37) |
| FEV_1_/FVC | 0.84 (0.11) | 0.82 (0.09) | 0.77 (0.11) | 0.79 (0.11) | 0.84 (0.12) | 0.80 (0.12) |
| DLCO % predicted | 28 (19) | 37 (18) | 31 (13) | 43 (22) | 34 (13) | 56 (20) |
| KCO % predicted | 51 (23) | 48 (20) | N/A | 67 (33) | 53 (19) | 70 (9) |
| FVC/DLCO ratio | 2.04 (0.81) | 1.92 (1.21) | 1.88 (0.47) | 1.71 (1.01) | 2.06 (1.04) | 1.48 (0.71) |
| 6-minute walk testing |  |  |  |  |  |  |
| 6-minute walk distance (m) | 276 (190) | 274 (169) | 139 (238) | 323 (156) | 270 (192) | 391 (204) |
| 6-minute walk % predicted (%) | 55 (38) | 55 (30) | 34 (47) | 67 (32) | 53 (28) | 58 (28) |
| Supplemental O2 use (N, %) | 57 (62.6%) | 9 (45.0%) | 4 (80.0%) | 19 (25.7%) | 22 (56.4%) | 3 (27.3%) |
| End-of-study peripheral O2 saturation (%) | 84 (11) | 86 (10) | 91 (17) | 88 (11) | 88 (12) | 90 (10) |
| End-of-study Borg dyspnea score | 4 (2) | 4 (3) | 5 (4) | 3 (2) | 4 (2) | 3 (1) |
| WSPH functional class | 3 (1) | 3 (1) | 3 (0) | 3 (1) | 3 (1) | 3 (1) |

e-Table 2. Univariate Cox proportional hazards ratios for the primary outcome, clinical worsening, and the secondary outcomes, hospitalization, physiologic worsening, and lung transplant or death.

|  | **Clinical**  **Worsening** | **Hospitalization** | **Physiologic**  **worsening** | **Lung transplant**  **or death** |
| --- | --- | --- | --- | --- |
|  | HR (95% CI) | HR (95% CI) | HR (95% CI) | HR (95% CI) |
| Age at PH diagnosis (years)* | 1.00 (0.99-1.01) | 1.00 (0.98-1.01) | 1.02 (1.00-1.03) | 1.01 (0.99-1.02) |
| Female sex | 0.80 (0.60-1.08) | 0.93 (0.64-1.34) | 0.73 (0.49-1.09) | 0.65 (0.45-0.92) |
| Patient-reported race |  |  |  |  |
| White | Ref | Ref | Ref | Ref |
| Black | 1.46 (1.11-1.92) | 1.87 (1.33-2.63) | 0.96 (0.66-1.40) | 1.17 (0.82-1.66) |
| Asian | 0.74 (0.35-1.60) | 0.79 (0.29-2.15) | 0.56 (0.18-1.78) | 0.98 (0.39-2.42) |
| Other | 1.58 (0.90-2.76) | 2.40 (1.30-4.45) | 1.92 (0.98-3.74) | 1.93 (1.02-3.65) |
| Hispanic ethnicity | 1.22 (1.05-1.42) | 1.15 (0.95-1.40) | 0.94 (0.75-1.20) | 1.66 (1.39-1.97) |
| Ever smoker | 0.91 (0.70-1.17) | 1.05 (0.77-1.43) | 0.91 (0.65-1.28) | 0.98 (0.72-1.35) |
| CTD diagnosis |  |  |  |  |
| Mixed connective tissue disease | 0.92 (0.54-1.57) | 1.05 (0.55-2.01) | 0.84 (0.41-1.74) | 0.76 (0.37-1.56) |
| Myositis | 1.23 (0.77-1.96) | 1.55 (0.91-2.63) | 1.17 (0.62-2.20) | 1.05 (0.59-1.87) |
| Rheumatoid arthritis | 2.31 (1.13-4.73) | 1.80 (0.73-4.44) | 2.02 (0.82-5.00) | 1.72 (0.76-3.93) |
| Scleroderma | 1.17 (0.43-3.16) | 1.54 (0.49-4.87) | 0.40 (0.06-2.87) | 1.18 (0.38-3.73) |
| Systemic lupus erythematosus | 1.07 (0.54-2.09) | 1.61 (0.79-3.37) | 1.55 (0.67-3.54) | 1.39 (0.61-3.17) |
| Undifferentiated CTD | 1.27 (0.77-2.10) | 1.09 (0.59-2.04) | 0.85 (0.41-1.76) | 0.51 (0.22-1.15) |
| Diffuse vs limited scleroderma | 1.12 (0.81-1.54) | 0.91 (0.60-1.36) | 0.98 (0.63-1.52) | 1.35 (0.92-2.00) |
| PH diagnosis prior to 2013 | 0.75 (0.57-0.98) | 0.64 (0.45-0.91) | 0.74 (0.51-1.08) | 1.06 (0.76-1.49) |
| WSPH group 3 PH vs group 1 | 1.21 (0.99-1.48) | 1.15 (0.89-1.49) | 1.27 (0.97-1.67) | 1.55 (1.20-2.00) |
| PFTs |  |  |  |  |
| Baseline FVC % predicted | 0.99 (0.98-1.00) | 0.99 (0.98-1.00) | 1.00 (0.99-1.01) | 0.98 (0.97-0.99) |
| FEV_1_ % predicted | 0.99 (0.99-1.00) | 0.99 (0.98-1.00) | 1.00 (0.99-1.01) | 0.98 (0.98-0.99) |
| FEV_1_/FVC | 1.00 (0.98-1.03) | 1.01 (0.98-1.03) | 0.95 (0.79-1.14) | 0.96 (0.82-1.11) |
| Baseline DLCO | 0.98 (0.97-0.99) | 0.97 (0.95-0.98) | 0.99 (0.98-1.00) | 0.96 (0.95-0.98) |
| Baseline DLCO/VA | 0.98 (0.97-0.99) | 0.97 (0.95-0.98) | 0.99 (0.98-1.00) | 0.97 (0.96-0.99) |
| Baseline FVC/DLCO | 1.42 (1.23-1.65) | 1.53 (1.31-1.79) | 1.36 (1.11-1.66) | 1.55 (1.29-1.87) |
| 6-minute walk testing |  |  |  |  |
| Baseline 6MWD % predicted | 0.99 (0.98-0.99) | 0.98 (0.98-0.99) | 1.00 (0.99-1.01) | 0.98 (0.97-0.99) |
| 6MWD O2 use | 2.24 (1.68-2.98) | 2.39 (1.68-3.40) | 1.34 (0.92-1.95) | 3.75 (2.54-5.54) |
| 6MWD final O2 sat | 0.95 (0.93-0.98) | 0.93 (0.91-0.96) | 0.98 (0.95-1.00) | 0.94 (0.91-0.96) |
| 6MWD Borg dyspnea score | 1.01 (0.99-1.03) | 1.01 (0.99-1.03) | 0.99 (0.95-1.04) | 1.02 (1.00-1.04) |
| RHC |  |  |  |  |
| RAP | 1.00 (0.96-1.04) | 1.01 (0.96-1.05) | 0.97 (0.92-1.02) | 1.01 (0.97-1.05) |
| mPAP | 1.01 (1.00-1.02) | 1.02 (1.00-1.03) | 1.00 (0.98-1.02) | 1.02 (1.01-1.04) |
| PCWP | 0.98 (0.94-1.02) | 1.02 (0.96-1.07) | 0.99 (0.94-1.05) | 0.97 (0.92-1.03) |
| CO | 1.00 (1.00-1.00) | 1.00 (1.00-1.00) | 1.00 (1.00-1.00) | 1.00 (1.00-1.00) |
| PVR | 1.00 (1.00-1.00) | 1.00 (1.00-1.00) | 1.00 (1.00-1.00) | 1.00 (1.00-1.00) |
| TTE |  |  |  |  |
| LVEF | 0.99 (0.97-1.01) | 0.98 (0.95-1.00) | 1.01 (0.98-1.04) | 0.99 (0.96-1.01) |
| Estimate RVSP | 1.01 (1.00-1.02) | 1.01 (1.01-1.02) | 1.00 (0.99-1.01) | 1.02 (1.01-1.03) |
| RV dysfunction | 1.93 (1.47-2.52) | 2.02 (1.46-2.81) | 1.40 (0.97-2.01) | 2.15 (1.54-3.01) |
| TAPSE | 0.47 (0.32-0.69) | 0.37 (0.23-0.59) | 0.81 (0.50-1.31) | 0.50 (0.31-0.82) |
| Log pro-BNP | 1.27 (1.17-1.39) | 1.29 (1.17-1.43) | 1.09 (0.98-1.22) | 1.38 (1.25-1.54) |
| WSPH functional class | 1.86 (1.47-2.35) | 2.05 (1.54-2.72) | 1.64 (1.20-2.25) | 2.61 (1.91-3.57) |

e-Table 3. Cox proportional hazards ratios for the primary and secondary outcomes comparing patients with scleroderma and non-scleroderma CTD for the parenchymal and vascular PH phenotypes. Models adjusted for age at the time of PH diagnosis, sex, race, PVR, and baseline FVC/DLCO

|  | **Scleroderma**  (N = 195) | **Non-scleroderma CTD**  (N = 80) |
| --- | --- | --- |
|  | **Adjusted HR** (95% CI) | **Adjusted HR** (95% CI) |
| Clinical worsening | 1.83 (1.25-2.68) | 1.60 (0.81-3.18) |
| Hospitalization | 1.56 (0.97-2.53) | 1.98 (0.92-4.30) |
| Physiologic worsening | 2.04 (1.25-3.32) | 1.44 (0.57-3.64) |
| Lung transplant or death | 2.05 (1.24-3.40) | 3.69 (1.37-9.97) |

e-Table 4. Cox proportional hazards ratios for the primary and secondary outcomes comparing patients with CTD-ILD who were ever exposed versus never exposed to PH medications and immunosuppression for the parenchymal and the vascular PH phenotypes. Models adjusted for age at the time of PH diagnosis, sex, race, PVR, and baseline FVC/DLCO.

|  | **Parenchymal**  (N = 134) | **Vascular**  (N = 141) |
| --- | --- | --- |
|  | **Adjusted HR** (95% CI) | **Adjusted HR** (95% CI) |
| PH medication |  |  |
| Clinical worsening | 1.04 (0.58-1.86) | 1.14 (0.56-2.32) |
| Hospitalization | 1.19 (0.59-2.41) | 1.04 (0.44-2.47) |
| Physiologic worsening | 0.94 (0.44-2.01) | 1.21 (0.46-3.16) |
| Lung transplant or death | 0.56 (0.29-1.09) | 0.40 (0.16-1.00) |
| Immunosuppression |  |  |
| Clinical worsening | 1.78 (0.92-3.45) | 1.01 (0.58-1.75) |
| Hospitalization | 6.30 (1.89-21.01) | 0.96 (0.48-1.92) |
| Physiologic worsening | 1.13 (0.50-2.53) | 1.60 (0.75-3.42) |
| Lung transplant or death | 1.15 (0.53-2.46) | 0.69 (0.34-1.40) |

e-Table 5. Cox proportional hazards ratios for the primary and secondary outcomes comparing patients with CTD-ILD and the parenchymal and the vascular PH phenotypes who were ever exposed to PH medications and immunosuppression. Models adjusted for age at the time of PH diagnosis, sex, race, PVR, and baseline FVC/DLCO.

|  | **Adjusted HR** (95% CI) |
| --- | --- |
| PH medication (N = 223) |  |
| Clinical worsening | 1.77 (1.24-2.53) |
| Hospitalization | 1.75 (1.13-2.70) |
| Physiologic worsening | 1.78 (1.12-2.82) |
| Lung transplant or death | 2.39 (1.46-3.90) |
| Immunosuppression (N = 220) |  |
| Clinical worsening | 2.00 (1.38-2.88) |
| Hospitalization | 2.20 (1.41-3.42) |
| Physiologic worsening | 1.72 (1.07-2.74) |
| Lung transplant or death | 2.60 (1.56-4.33) |

e-Table 6. Comparisons of the model-fit (Akaike’s information criteria) and discriminant ability (c-statistics) of the WSPH groups and CT-based phenotypes based on adjusted Cox proportional hazards regression.

|  | **AIC** | | **c-statistics** | | |
| --- | --- | --- | --- | --- | --- |
|  | WSPH Group | CT-based Phenotype | WSPH Group | CT-based Phenotype | P-value |
| Clinical worsening | 1479 | 1469 | 0.55 | 0.62 | 0.11 |
| Hospitalization | 997 | 991 | 0.50 | 0.57 | 0.04 |
| Physiologic worsening | 929 | 922 | 0.50 | 0.53 | 0.29 |
| Death or lung transplantation | 875 | 865 | 0.60 | 0.63 | 0.38 |
